# The adenosine A_2B_ receptor is involved in anion secretion in human pancreatic duct Capan-1 epithelial cells

**DOI:** 10.1007/s00424-016-1806-9

**Published:** 2016-03-11

**Authors:** M. Hayashi, A. Inagaki, I. Novak, H. Matsuda

**Affiliations:** Department of Physiology, Kansai Medical University, 2-5-1 Shimmachi, Hirakata, 573-1010 Japan; Medical Research Project, Institute of Biomedical Sciences, Tokushima University Graduate School, 3-18-15 Kuramoto-cho, Tokushima, 770-8503 Japan; Section for Cell Biology and Physiology, Department of Biology, University of Copenhagen, August Krogh Building, Universitetsparken 13, 2100 Copenhagen Ø, Denmark

**Keywords:** Adenosine receptor, CFTR, Cl^−^ channel, Duct, Pancreas

## Abstract

Adenosine modulates a wide variety of biological processes via adenosine receptors. In the exocrine pancreas, adenosine regulates transepithelial anion secretion in duct cells and is considered to play a role in acini-to-duct signaling. To identify the functional adenosine receptors and Cl^−^ channels important for anion secretion, we herein performed experiments on Capan-1, a human pancreatic duct cell line, using open-circuit Ussing chamber and gramicidin-perforated patch-clamp techniques. The luminal addition of adenosine increased the negative transepithelial potential difference (*V*_te_) in Capan-1 monolayers with a half-maximal effective concentration value of approximately 10 μM, which corresponded to the value obtained on whole-cell Cl^−^ currents in Capan-1 single cells. The effects of adenosine on *V*_te_, an equivalent short-circuit current (*I*_sc_), and whole-cell Cl^−^ currents were inhibited by CFTRinh-172, a cystic fibrosis transmembrane conductance regulator (CFTR) Cl^−^ channel inhibitor. The adenosine A_2B_ receptor agonist, BAY 60-6583, increased *I*_sc_ and whole-cell Cl^−^ currents through CFTR Cl^−^ channels, whereas the A_2A_ receptor agonist, CGS 21680, had negligible effects. The A_2B_ receptor antagonist, PSB 603, inhibited the response of *I*_sc_ to adenosine. Immunohistochemical analysis showed that the A_2A_ and A_2B_ receptors colocalized with Ezrin in the luminal membranes of Capan-1 monolayers and in rat pancreatic ducts. Adenosine elicited the whole-cell Cl^−^ currents in guinea pig duct cells. These results demonstrate that luminal adenosine regulates anion secretion by activating CFTR Cl^−^ channels via adenosine A_2B_ receptors on the luminal membranes of Capan-1 cells. The present study endorses that purinergic signaling is important in the regulation of pancreatic secretion.

## Introduction

The pancreas plays a pivotal role in digestion. Pancreatic acini secrete digestive enzymes, and ducts secrete a HCO_3_^−^-rich pancreatic juice that neutralizes acid chyme in the duodenum. The generally accepted model for HCO_3_^−^ transport involves Cl^−^–HCO_3_^−^ exchangers that operate in parallel with cAMP-activated Cl^−^ channels [cystic fibrosis transmembrane conductance regulator (CFTR)] and Ca^2+^-activated Cl^−^ channels, such as TMEM16A/ANO1, on the luminal membranes of duct cells [46, 50].

Extracellular adenosine has been shown to modulate a wide variety of biological processes via cell surface adenosine receptors [6, 10]. There are four known adenosine receptors denoted adenosine A_1_, A_2A_, A_2B_, and A_3_ receptors. A_2A_ and A_2B_ receptors generally increase, whereas A_1_ and A_3_ receptors decrease cAMP levels [11]. Previous studies reported that adenosine activated A_1_, A_2A_, A_2B_, and A_3_ receptors with half-maximal effective concentration (EC_50_) values of 0.1, 0.3, 15, and 0.3 μM, respectively [10].

In pancreatic ducts, adenosine is produced by the hydrolysis of ATP, which is secreted from acini in response to cholinergic and hormonal stimuli [15, 16, 39, 52]. Previous studies by Novak and coworkers have demonstrated that adenosine activates Cl^−^ conductance in rat pancreatic duct cells and induce Cl^−^ efflux in a human duct cell line (PANC-1) using a patch-clamp analysis and Cl^−^-sensitive fluorophore, respectively [32]. In addition, adenosine induced anion secretion that was larger on the luminal side compared to the basolateral side in a human pancreatic duct cell line (Capan-1) monolayer [47]. Rat pancreatic ducts and human duct cell lines (PANC-1 and CFPAC-1) were found to express adenosine A_1_, A_2A_, A_2B_, and A_3_ receptors, with the adenosine A_2A_ and A_2B_ receptors being the most abundant at the messenger RNA (mRNA) level [32]. Furthermore, adenosine A_2A_ receptors were detected on the luminal membranes of rat ducts and plasma membrane of PANC-1 cells [32]. Therefore, adenosine and ATP are regarded as acini-to-duct messengers that stimulate ductal secretion [32, 38]. However, the molecular basis of functional adenosine receptors and the intracellular mechanism of ductal secretion via adenosine remain inconclusive.

The aim of the present study was to identify functional adenosine receptors and Cl^−^ channels using pharmacological and electrophysiological tools. Capan-1 cells have been shown to conserve most of the properties of duct cells, including the functional expression of CFTR Cl^−^ channels and Ca^2+^-activated Cl^−^ channels, and are, thus, widely used as an epithelial model of human pancreatic ducts [7, 18, 24, 25, 31, 44–47]. ATP and UTP were shown to regulate CFTR Cl^−^ channels, Ca^2+^-activated Cl^−^ channels (TMEM16A/ANO1), and Ca^2+^-activated K^+^ channels (K_Ca_3.1) via purinergic receptors [18, 46]. In the present study, we demonstrate that luminal adenosine regulates transepithelial anion secretion by activating CFTR Cl^−^ channels via adenosine A_2B_ receptors on the luminal membranes of Capan-1 cells. Furthermore, we show that luminal adenosine activates Cl^−^ conductance in native duct cells from guinea pig.

## Methods

### Cell culture

Capan-1 cells were grown to confluent monolayers and mounted in Ussing chambers for open-circuit recordings, as described in detail previously [47]. Briefly, Capan-1 cells (#HTB-79; ATCC) were grown in Iscove’s modified Dulbecco’s medium with Glutamax and 20 % FBS (Gibco) [26]. Regarding Ussing chamber studies, cells were grown on membranes (Snapwell, Costar 3801; Corning) in 37 °C and 5 % CO_2_ for 7–28 days until confluent monolayers were formed. Cells from passages 23 to 30 were used in this study.

### Open-circuit Ussing chamber measurements

Capan-1 monolayers were mounted in mini-Ussing chambers (model P2300, Easymount Chamber System; Physiologic Instruments) and electrophysiological parameters were recorded, as described in detail previously [22, 47]. Briefly, the luminal and basolateral compartments were filled with a solution containing the following (in mM): 115 NaCl, 5 KCl, 1 CaCl_2_, 1 MgCl_2_, 25 NaHCO_3_, 10 HEPES (pH 7.4, adjusted with NaOH), and 10 D-glucose. The solution was equilibrated with 5 % CO_2_ in O_2_. The temperature was kept constant at 37 °C during all experiments. The transepithelial potential difference (*V*_te_) was monitored using 3 M KCl/agar and Ag/AgCl cartridge electrodes connected to a current-clamp amplifier (CEZ-9100; Nihon Kohden). Current pulses of 18 μA/cm^2^ were applied at 5-s intervals, and transepithelial resistance (*R*_te_) was calculated. The equivalent short-circuit current (*I*_sc_) was calculated from the *V*_te_ and *R*_te_ values. *V*_te_ is expressed as luminal with respect to basolateral side. *I*_sc_ is referred to as positive for current flowing across the epithelium from luminal to basolateral side. Data were transferred to digital signals through PowerLab 16/30 and were recorded using Chart 7 (ADInstruments).

Adenosine was obtained from Sigma-Aldrich. 4-[[4-Oxo-2-thioxo-3-[3-trifluoromethyl)phenyl]-5-thiazolidinylidene]methyl]benzoic acid (CFTRinh-172), niflumic acid, 5-nitro-2-(3-phenylpropylamino)benzoic acid (NPPB), and 2-[(5-ethyl-1,6-dihydro-4-methyl-6-oxo-2-pyrimidinyl)thio]-N-[4-(4-methoxyphenyl)-2-thiazolyl]acetamide (T16Ainh-A01) were obtained from Santa Cruz Biotechnology, Cayman Chemical, Enzo Life Sciences, and Merck Millipore, respectively. 2-[6-Amino-3,5-dicyano-4-[4-(cyclopropylmethoxy)phenyl]pyridin-2-ylsulfanyl]acetamide (BAY 60-6583), 4-[2-[[6-Amino-9-(N-ethyl-β-D-ribofuranuronamidosyl)-9H-purin-2-yl]amino]ethyl]benzenepropanoic acid (CGS 21680), and 8-[4-[4-(4-chlorophenzyl)piperazide-1-sulfonyl)phenyl]]-1-propylxanthine (PSB 603) were obtained from Tocris Bioscience.

### Patch-clamp whole-cell recording

Gramicidin-perforated patch techniques were used [19]. Gramicidin D (Sigma-Aldrich) was dissolved in DMSO at 20 mg/ml and then diluted to a final concentration of 0.1 mg/ml in a standard KCl-rich pipette solution containing the following (in mM): 150 KCl and 10 HEPES; pH was adjusted to 7.4 with KOH. The pipette tip was filled with the gramicidin-free pipette solution by a brief immersion. The pipette was then back-filled with the gramicidin-containing pipette solution. Patch pipettes (G-1.5; Narishige) had a resistance of 3–4 MΩ when filled with the pipette solution. A standard bathing solution contained the following (in mM): 150 NaCl, 1 CaCl_2_, and 5 HEPES; pH was adjusted to 7.4 with NaOH. The membrane potential was corrected for the liquid junction potential at the tip of the patch pipette in the bathing solution and for that at the tip of the indifferent reference electrode filled with bathing solution and placed in the bath. Experiments were conducted at 23–30 °C. The whole-cell current was recorded using the EPC 800 patch-clamp amplifier (HEKA). The amplifier was driven by Clampex 9 (Axon) in order to allow the delivery of a voltage-ramp protocol with concomitant digitization of the current. Gramicidin-perforated patch recording was started after stabilization of the capacitive current. The capacitance transient current was compensated by the amplifier. Whole-cell capacitance and series resistance (*R*_s_) were 13.5 ± 0.9 pF and 47.8 ± 4.5 MΩ (*n* = 28), respectively, in experiments using Capan-1 single cells. Since *R*_s_ was not electronically compensated for, the conductance of currents was underestimated as a result of the voltage decrease across *R*_s_, and the potential reported here was not corrected for *R*_s_. The voltage decrease was at most 20 mV. The whole-cell current was filtered at 1 kHz with an internal four-pole Bessel filter, sampled at 2 kHz, and transferred to digital signals through Digidata 1322A (Axon). A subsequent current analysis was performed using Clampfit 9 (Axon).

### Immunolocalization

Immunolocalization was performed on Capan-1 monolayers and the rat pancreas. The pancreas was obtained from male Wistar rats (*n* = 3). Protocols involving the handling of animals were approved by the Animal Experimentation Committee, Kansai Medical University. Animals were killed by cervical dislocation. Detailed methods for immunohistochemistry are described elsewhere [18]. Briefly, the rat pancreas was cut into small pieces and fixed with 4 % paraformaldehyde in PBS for 24 h. A confluent Capan-1 monolayer was fixed with 4 % paraformaldehyde for 15 min and permeabilized with 0.2 % Triton X-100 in PBS for 10 min. Autofluorescence was blocked in 0.1 M Tris-glycine. Nonspecific binding was blocked with 2 % normal donkey serum in PBS. Preparations were subsequently incubated with primary antibodies for the adenosine A_2A_ receptor (1:100, sc-13937; Santa Cruz Biotechnology), adenosine A_2B_ receptor (1:800, AAR-003; Alomone), or cytokeratin 20 (1:100, EPR1622Y, ab76126; Abcam) with Ezrin (1:200 to 1:400, clone 3C12, MS-661; Lab Vision) and PECAM-1 (platelet endothelial cell adhesion molecule-1, 1:400, sc-1506; Santa Cruz Biotechnology) in immunoreaction enhancer solution (Can Get Signal immunostain; Toyobo) overnight at 4 °C. Secondary antibodies conjugated to Alexa488, Alexa568, or Alexa647 (1:400; Molecular Probes) were added for 30 min. In the controls, the primary antibodies were omitted and scanning was performed using the same settings. Nuclei were stained with 4′,6-diamidino-2-phenylindole (DAPI) at 1 μg/ml. Fluorescence was observed with a confocal laser scanning microscope (LSM510 META; Carl Zeiss).

### Preparation of pancreatic duct cells from guinea pig

Female Hartley guinea pigs (290–440 g, *n* = 10) were killed by cervical dislocation in accordance with the protocols approved by the Animal Experimentation Committee, Kansai Medical University. Pancreatic ducts were isolated by enzymatic digestion and microdissection from the pancreas as previously described [18, 32]. Pancreas was removed and digested with collagenase (type IV, 124 U/ml; Worthington) and trypsin inhibitor (0.01 %; Sigma) in Tyrode solution at 37 °C for 1 h with vigorous shaking. The Tyrode solution contained the following (in mM): 140 NaCl, 0.33 NaH_2_PO_4_, 5.4 KCl, 1.8 CaCl_2_, 0.5 MgCl_2_, 5 HEPES, and 5.5 D-glucose; pH was adjusted to 7.4 with NaOH. Interlobular and intralobular ducts (outside diameter of 30–60 μm) were microdissected under a stereomicroscope. The ducts were washed in Tyrode solution and then placed on coverslips pretreated with Cell-Tak (BD Biosciences). In order to allow patch-clamp access to the luminal membranes of lining epithelial cells, the ducts were split open by patch pipettes.

### Statistics

Data are shown as means ± SEM. A one-way analysis of variance or Student’s paired *t* test was applied, and *P* < 0.05 was considered significant. Data were analyzed in Igor or Microsoft Excel.

## Results

### Effects of luminal adenosine on transepithelial anion secretion in Capan-1 monolayers

**Fig. 1:**
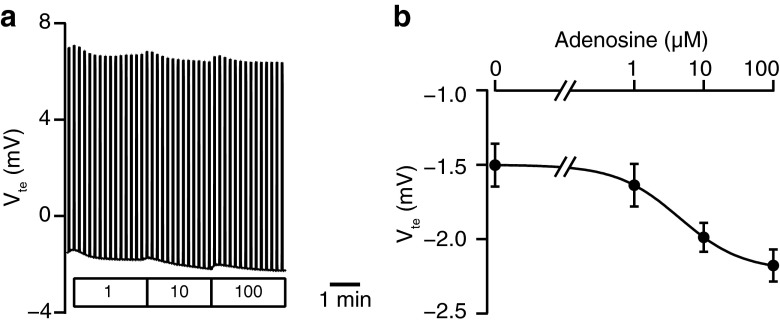
Effects of adenosine on the transepithelial potential difference (*V*_te_) of Capan-1 monolayers. **a** The *V*_te_ of a monolayer is shown as a function of time; current pulses were used to determine transepithelial resistance (*R*_te_). The representative trace demonstrates the increase observed in negative *V*_te_ in response to luminal adenosine (1–100 μM) in a concentration-dependent manner. **b** Concentration-response curve for adenosine. The *solid line* is the fit by the Hill equation (*n* = 4)

**Fig. 2:**
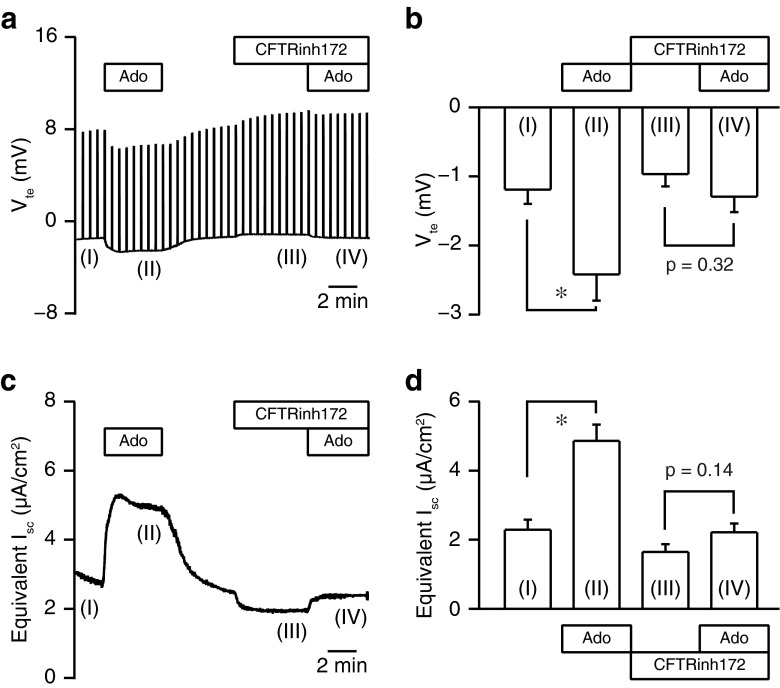
Effects of CFTRinh-172 on the adenosine stimulation in Capan-1 monolayers. **a** The representative trace demonstrates the increase observed in negative *V*_te_ in response to adenosine (*Ado*; 100 μM) (phase II) and the inhibition by CFTRinh-172 (20 μM) (phase IV) on the luminal membrane. **b** Summary of *V*_te_ recordings (*n* = 5). Numbers (I, II, III, and IV) correspond to the control and test periods of the experiment depicted in **a**. *P < 0.05. **c** An equivalent short-circuit current (*I*_sc_) trace from the same experiment as shown in **a. d** Summary of the effects of adenosine and CFTRinh-172 on *I*_sc_ (*n* = 5)

**Fig. 3:**
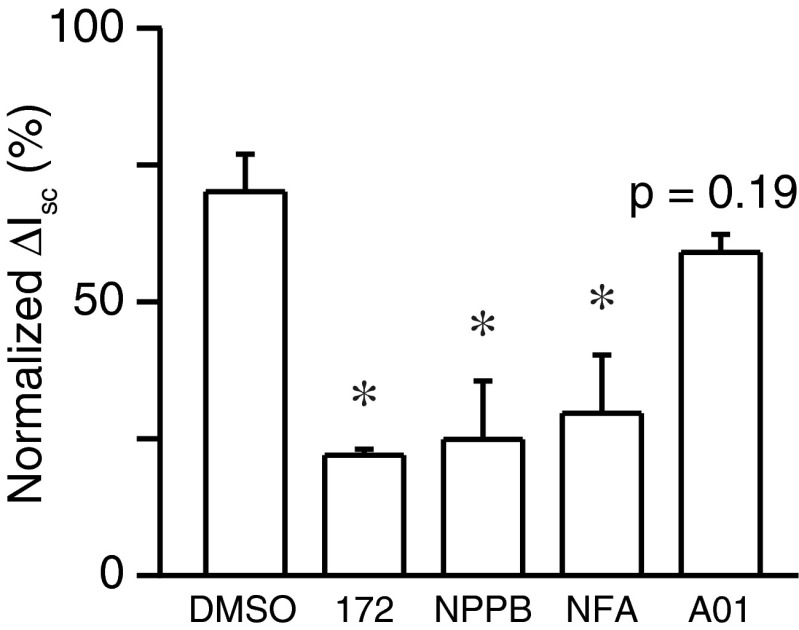
Summary of the effects of Cl^−^ channel blockers on changes in the short-circuit current (Δ*I*_sc_) of Capan-1 monolayers stimulated with adenosine (100 μM). Δ*I*_sc_ during the second stimulation in the presence of Cl^−^ channel blockers was expressed as a percentage of that during the first stimulation. DMSO (vehicle control; 0.1 %), CFTRinh-172 (172; 20 μM), NPPB (100 μM), niflumic acid (*NFA*; 100 μM), and T16Ainh-A01 (A01; 10 μM) (*n* = 5–11). *P < 0.05

In order to determine whether adenosine regulated transepithelial anion secretion in pancreatic duct cells, we measured the electrophysiological parameters of the Capan-1 monolayer in Ussing chambers. In the present series of experiments, the Capan-1 monolayer displayed a resting transepithelial resistance (*R*_te_) of 400 ± 14 Ω cm^2^, transepithelial potential difference (*V*_te_) of −1.14 ± 0.05 mV, and equivalent short-circuit current (*I*_sc_) of 2.42 ± 0.08 μA/cm^2^ (*n* = 60). Figure 1a shows a representative original *V*_te_ recording. The luminal addition of adenosine increased negative *V*_te_, which indicated either transepithelial anion secretion or cation absorption, in a concentration-dependent manner. The EC_50_ value for the effects of adenosine was estimated at 11.6 ± 6.5 μM with a Hill coefficient of 1.3 ± 0.3 (Fig. 1b; *n* = 4). The response to adenosine (100 μM) was relatively reproducible in repeated applications. The response to adenosine on *V*_te_ was reduced by NPPB (100 μM) or niflumic acid (100 μM), nonselective Cl^−^ channel blockers applied to the luminal side (*n* = 5; not shown). The luminal addition of the CFTR Cl^−^ channel inhibitor (20 μM CFTRinh-172) inhibited the response to adenosine (Fig. 2a, b; *n* = 5). The calculated *I*_sc_ also showed that adenosine elicited transepithelial anion secretion and the response to the second stimulation with adenosine was inhibited by CFTRinh-172 (Fig. 2c, d). Figure 3 summarizes the effects of Cl^−^ channel blockers on increases in *I*_sc_ (Δ*I*_sc_) stimulated by adenosine. We normalized Δ*I*_sc_ during the second stimulation in the presence of Cl^−^ channel blockers to Δ*I*_sc_ during the first stimulation and compared their effects. Δ*I*_sc_ was not inhibited by T16Ainh-A01 (10 μM), a TMEM16A/ANO1 channel inhibitor (*n* = 9).

**Fig. 4:**
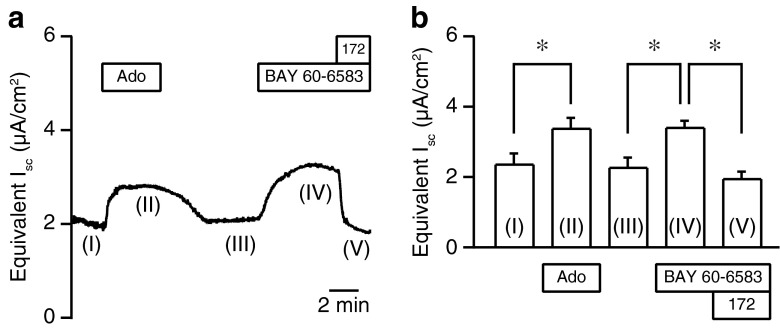
BAY 60-6583 stimulated *I*_sc_ in Capan-1 monolayers. **a** The representative trace demonstrates the increase in *I*_sc_ in response to BAY 60-6583 (10 μM) (phase IV) and the inhibition by CFTRinh-172 (172; 20 μM) (phase V) on the luminal membrane. *Ado* adenosine (100 μM). **b** Summary of equivalent *I*_sc_ recordings (*n* = 6). Numbers (I, II, III, IV, and V) correspond to the numbers in **a**. *P < 0.05

**Fig. 5:**
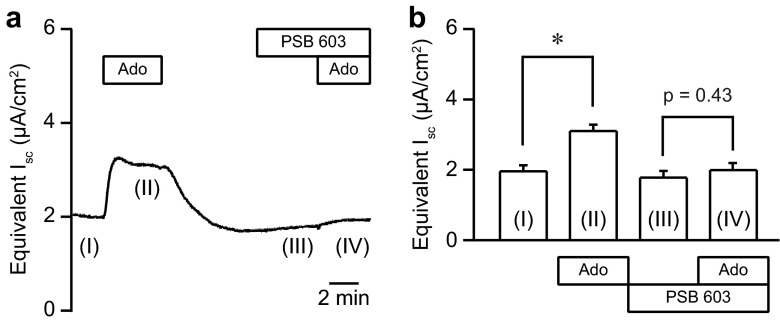
PSB 603 inhibited the adenosine stimulation of *I*_sc_ in Capan-1 monolayers. **a** The representative *I*_sc_ trace demonstrates the inhibition by PSB 603 (1 μM) in response to adenosine (100 μM) (phase IV). **b** Summary of equivalent *I*_sc_ recordings (*n* = 9). Numbers (I, II, III, and IV) correspond to the numbers in **a**. *P < 0.05

Specific adenosine receptor agonists were tested to identify functional adenosine receptors in duct cells [10]. The luminal addition of CGS 21680 (10 μM), an adenosine A_2A_ receptor agonist, had a negligible effect on *I*_sc_ in the Capan-1 monolayer: 2.01 ± 0.28 μA/cm^2^ in the control and 2.10 ± 0.30 μA/cm^2^ with CGS 21680 (*P* = 0.83, *n* = 6; not shown). On the other hand, BAY 60-6583 (10 μM), an adenosine A_2B_ receptor agonist, increased *I*_sc_ from 2.28 ± 0.27 to 3.41 ± 0.19 μA/cm^2^, and CFTRinh-172 decreased *I*_sc_ to 1.96 ± 0.20 μA/cm^2^ (Fig. 4; *n* = 6). Furthermore, PSB 603 (1 μM), an adenosine A_2B_ receptor antagonist, inhibited the response of *I*_sc_ to adenosine (Fig. 5; *n* = 9). These results indicate that the adenosine A_2B_ receptor mediates increases in anion transport through CFTR Cl^−^ channels on the luminal membranes of the Capan-1 monolayer.

### Whole-cell Cl^−^ conductance in Capan-1 single cells with gramicidin-perforated patch methods

**Fig. 6:**
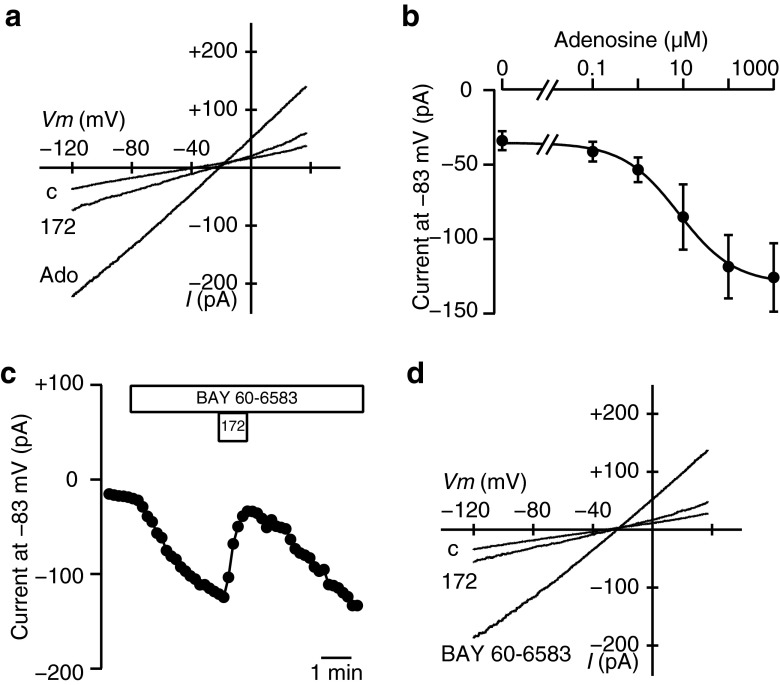
Effects of adenosine and BAY 60-6583 on the whole-cell current in Capan-1 single cells. **a** Representative current-voltage relationships for the whole-cell current. The current was elicited by a voltage ramp from −123 to +37 mV with a rate of 0.2 V/s. Adenosine (*Ado*) increased the current in the NaCl-rich bathing solution (*c*). Thereafter, the current induced by adenosine decreased by CFTRinh-172 (172). **b** Concentration-response curve for adenosine at −83 mV. The *solid line* is the fit by the Hill equation (*n* = 5). **c** The representative trace demonstrates the increase observed in the inward current at −83 mV in response to BAY 60-6583 (10 μM) and the inhibition by CFTRinh-172 (172; 20 μM). **d** Representative current-voltage relationships for the whole-cell current from the same recording shown in **c**. BAY 60-6583 increased the current in the NaCl-rich bathing solution (*c*). Thereafter, the current induced by BAY 60-6583 decreased by CFTRinh-172 (172)

We confirmed the results obtained so far using patch-clamp methods. In order to verify that adenosine activated CFTR Cl^−^ channels, we measured whole-cell currents in Capan-1 single cells using gramicidin-perforated patch techniques. The application of 100 μM adenosine increased slope conductance in a voltage range between −103 and −63 mV from 1.03 ± 0.19 to 2.61 ± 0.48 nS, and this was inhibited to 1.72 ± 0.22 nS by 20 μM CFTRinh-172 (Fig. 6a; *n* = 8). The EC_50_ value for the effects of adenosine was estimated at 9.2 ± 5.3 μM with a Hill coefficient of 1.1 ± 0.2 (Fig. 6b), corresponding to the EC_50_ value on *V*_te_ in Capan-1 monolayers. Consistent with the results obtained from measurements of *I*_sc_, the application of 10 μM CGS 21680 did not significantly increase slope conductance from 0.63 ± 0.09 to 1.21 ± 0.31 nS (*P* = 0.14, *n* = 6; not shown). The application of 10 μM BAY 60-6583 induced a sustained inward current at −83 mV, and this was reversibly inhibited by 20 μM CFTRinh-172 (Fig. 6c; *n* = 13). The current response to BAY 60-6583 was observed in 68 % (13 out of 19) of the cells tested. BAY 60-6583 increased slope conductance from 0.45 ± 0.05 to 1.49 ± 0.42 nS, and this was inhibited to 0.58 ± 0.10 nS by CFTRinh-172 (Fig. 6d; *n* = 13). When chloride was substituted with equimolar glutamate in the bathing solution, the reversal potential of the current-voltage curve shifted from −30.6 ± 2.8 to −4.5 ± 9.3 mV (*n* = 9; not shown), indicating that membrane conductance was chloride selective. Furthermore, the inward current induced by BAY 60-6583 was also observed in a bathing solution in which sodium was replaced with *N*-methyl-d-glucamine (*n* = 5, not shown), suggesting that the current through sodium-permeable cation channels was negligible.

### Immunolocalization of adenosine receptors in pancreatic duct cells

**Fig. 7:**
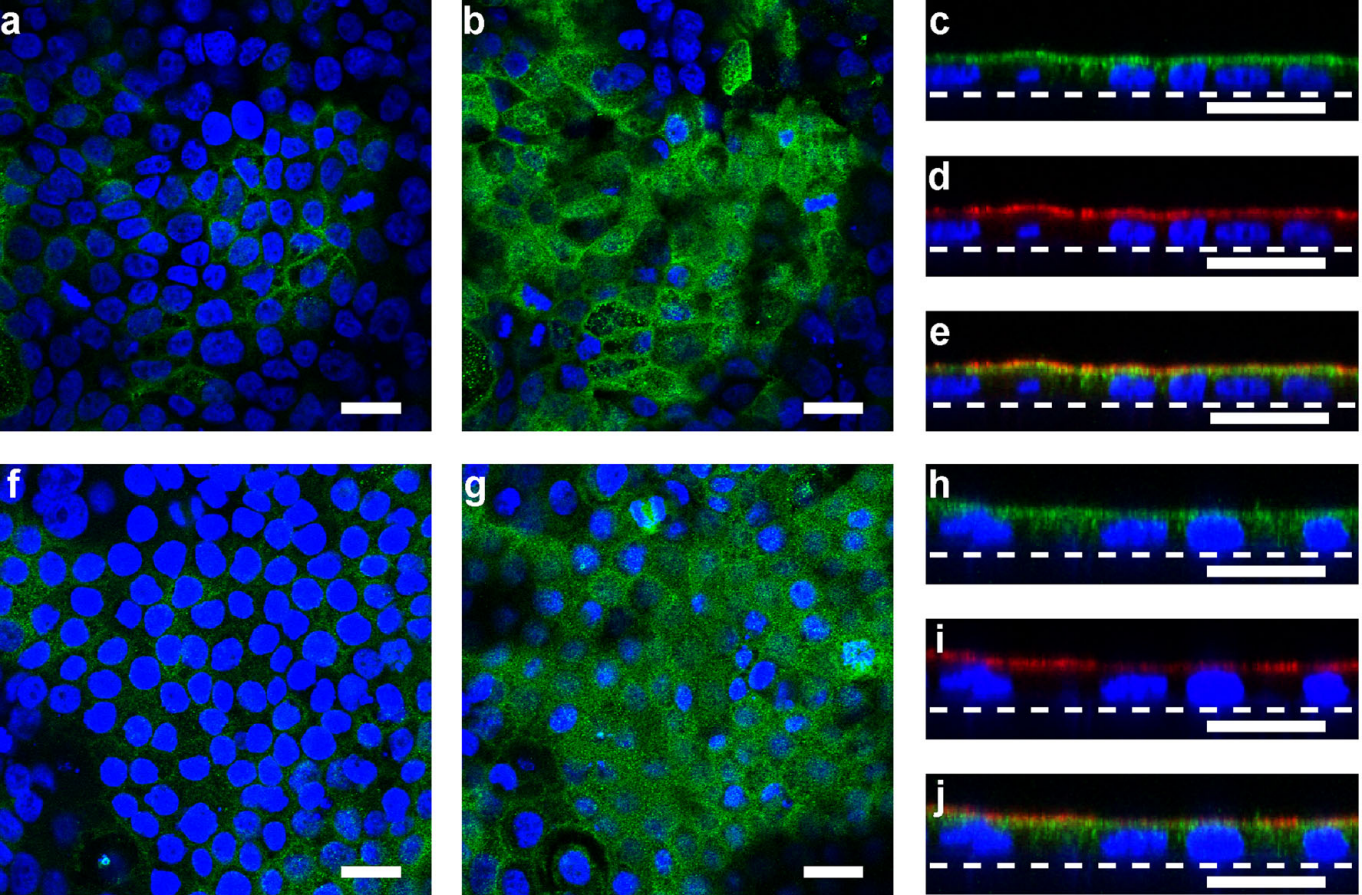
Immunolocalization of adenosine A_2A_ (**a**–**e**) and A_2B_ (**f**–**j**) receptors with Ezrin staining in Capan-1 monolayers. Fluorescence images of adenosine A_2A_ receptors on the basolateral (**a**) and luminal (**b**) membranes in the Capan-1 monolayer. **c** Z-scan image of the same sample in **a** and **b. d** Fluorescence image of Ezrin. **e** Overlay image of **c** and **d**. *Broken lines* indicate the position of the permeable membrane. Fluorescence images of adenosine A_2B_ receptors on the basolateral (**f**) and luminal (**g**) membranes in the Capan-1 monolayer. Z-scan images of adenosine A_2B_ receptors (**h**), Ezrin (**i**), and overlay (**j**). DAPI was used to stain nuclei (*blue*). *Bars* = 20 μm

**Fig. 8:**
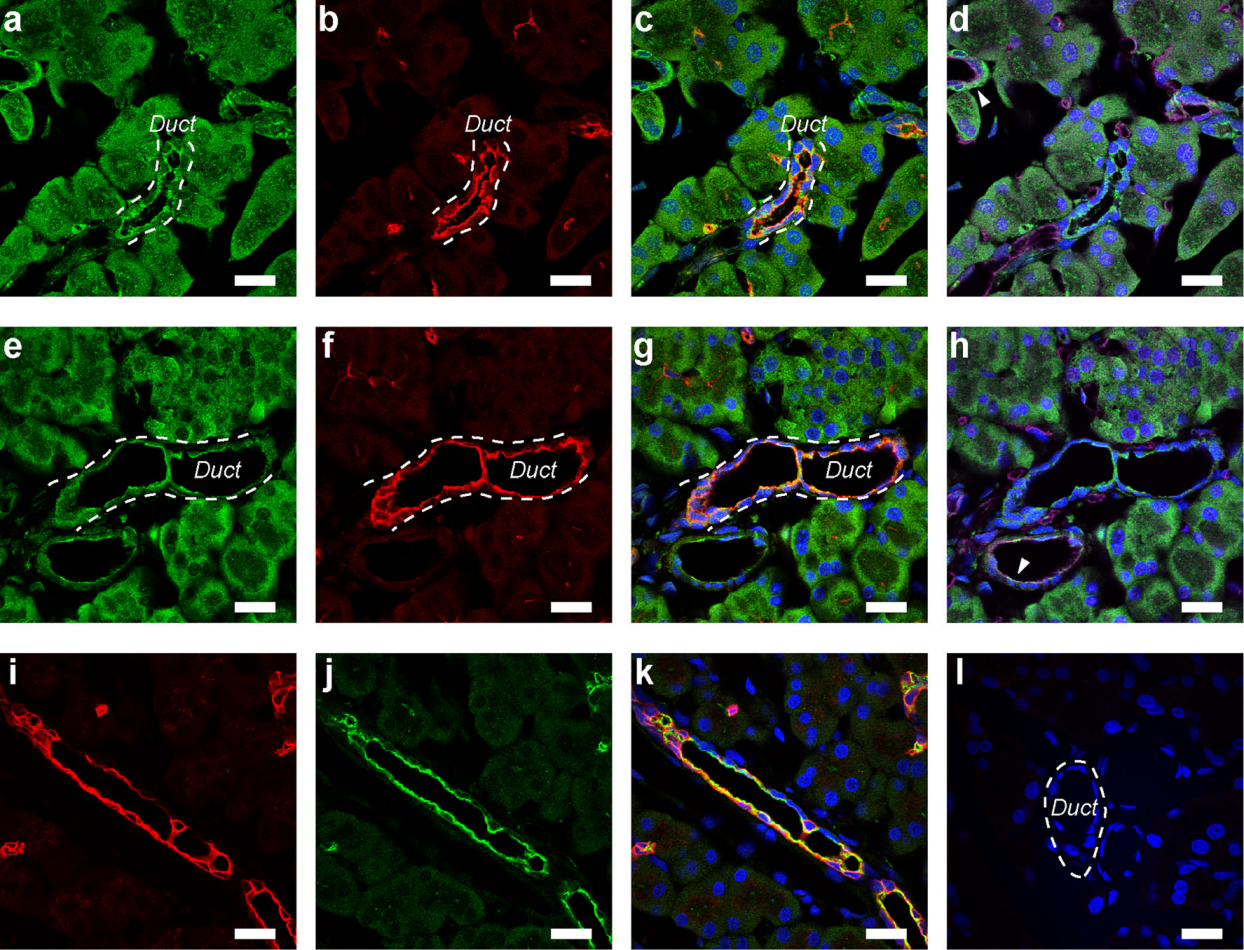
Immunolocalization of adenosine receptors in the rat pancreas. **a** Fluorescence of adenosine A_2A_ receptors on the luminal membranes of duct cells. The duct is indicated by *broken lines*. **b** Fluorescence image of Ezrin. **c** Overlay image of **a** and **b. d** Overlay of **a** and fluorescence image of a blood vessel marker (*purple*: PECAM-1) in the same sample. *Arrowhead* shows a blood vessel**. e** Fluorescence of adenosine A_2B_ receptors on the luminal membranes of a duct (*broken lines*). Fluorescence images of Ezrin (**f**) and overlay (**g**). **h** The overlay image shows the green fluorescence of adenosine A_2B_ receptors on a blood vessel (*arrowhead*). Fluorescence images of cytokeratin 20 (**i**), Ezrin (**j**), and overlay (**k**). **l** Control image of the rat pancreas, in which primary antibodies were omitted. The *broken line* indicates a duct. DAPI was used to stain nuclei (*blue*). *Bars* = 20 μm

The immunolocalization of adenosine receptors was performed using Capan-1 monolayers and paraffin sections of the rat pancreas. Immunofluorescence ascribed to the adenosine A_2A_ and A_2B_ receptors was colocalized with Ezrin, an A-kinase anchoring protein, in the luminal membranes of Capan-1 monolayers (Fig. 7). Notably, the adenosine A_2A_ receptors were expressed in the Capan-1 monolayers even though CGS 21680 had a negligible effect on *I*_sc_. In the rat pancreas, A_2A_ immunofluorescence was detected on the luminal membranes of duct cells (Fig. 8a), as reported previously [32]. Adenosine A_2A_ receptors were colocalized with Ezrin in the luminal membranes (Fig. 8b, c). Furthermore, adenosine A_2B_ receptors were colocalized with Ezrin in the luminal membranes of duct cells (Fig. 8e–g). The signal for Ezrin was detected on the luminal membranes of duct cells in which cytokeratin 20, a duct marker [4], was expressed (Fig. 8i–k). Additionally, the signal for adenosine A_2A_ and A_2B_ receptors was detected on the endothelial cells of blood vessels (Fig. 8d, h).

### Whole-cell Cl^−^ conductance in pancreatic duct cells from guinea pig

**Fig. 9:**
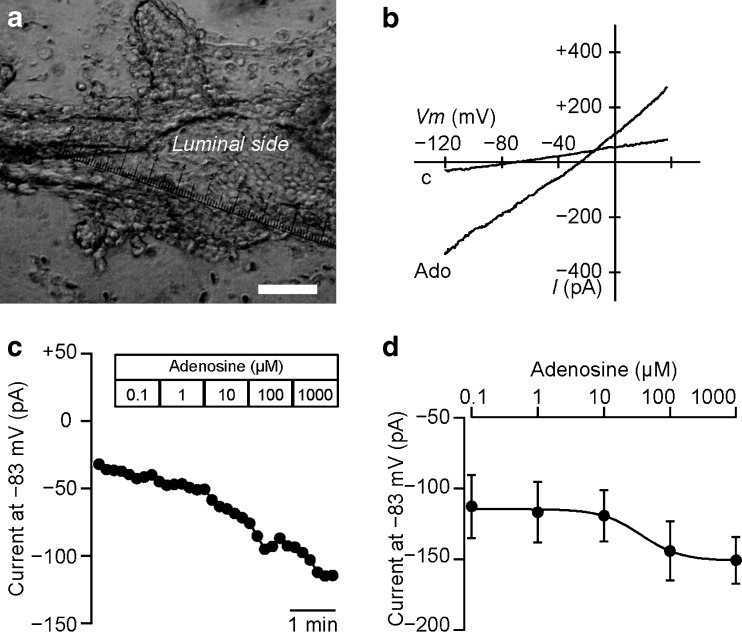
Effects of adenosine on the whole-cell current in pancreatic duct cell from guinea pig. **a** An isolated interlobular duct of the guinea pig pancreas. The duct, which has an outside diameter of about 50 μm, is split open to allow patch-clamp access to the luminal membranes of lining epithelial cells. The duct is held by the pipette on the left side. *Bar* = 50 μm. **b** Representative current-voltage relationships for the whole-cell current. The current was elicited by a voltage ramp from −123 to +37 mV with a rate of 0.2 V/s. Adenosine (*Ado*; 100 μM) increased the current in the NaCl-rich bathing solution (*c*). **c** The representative trace demonstrates the increase observed in the inward current at −83 mV in response to adenosine (0.1–1000 μM) in a concentration-dependent manner. **d** Concentration-response curve for adenosine at −83 mV. The *solid line* is the fit by the Hill equation (*n* = 5)

In order to demonstrate the luminal stimulatory effect of adenosine on native pancreatic ducts, we measured whole-cell currents in guinea pig duct cells using gramicidin-perforated patch techniques. These ducts were split open to allow the patch pipettes and bathing solution to access to the luminal membranes of lining epithelial cells (Fig. 9a). The application of 100 μM adenosine significantly increased slope conductance in a voltage range between −103 and −63 mV from 2.25 ± 0.57 to 3.72 ± 0.63 nS (Fig. 9b; *n* = 13). Adenosine induced a sustained inward Cl^−^ current at −83 mV in a concentration-dependent manner (Fig. 9c). The concentration-response curve was fitted with a Hill equation and the EC_50_ value was 21.2 ± 11.7 μM with a Hill coefficient of 1.0 ± 0.3 (Fig. 9d; *n* = 5). Recent studies have shown that ethanol affects the function of CFTR Cl^−^ channels in pancreatic epithelial cells [23]. The application of ethanol did not show significant effects on the adenosine-stimulated conductance in a voltage range between −103 and −63 mV from 2.49 ± 0.30 to 2.85 ± 0.30 at 1 mM and 3.08 ± 0.35 nS at 10 mM within 2 min in guinea pig duct cells (*P* = 0.34 and 0.20, respectively, *n* = 7; not shown).

## Discussion

In the present study, we demonstrated that the luminal adenosine A_2B_ receptor regulated the CFTR Cl^−^ channels necessary for anion secretion in Capan-1 cells. This conclusion was based on the following major results: the luminal addition of adenosine elicited transepithelial anion transport through CFTR Cl^−^ channels in Capan-1 monolayers; the adenosine A_2B_ receptor agonist activated anion transport; the adenosine response was inhibited by the adenosine A_2B_ receptor antagonist; the adenosine A_2B_ receptor agonist activated CFTR Cl^−^ channels in Capan-1 single cells; the adenosine A_2B_ receptors colocalized with Ezrin in the luminal membranes of Capan-1 monolayers and rat pancreatic ducts; and adenosine elicited the whole-cell Cl^−^ currents in pancreatic duct cells from guinea pig.

Adenosine A_2B_ receptors primarily signal via G_s_ proteins, resulting in the activation of adenylyl cyclase, an increase in cAMP production, activation of a membrane-associated isoform of protein kinase A (type II PKA), and subsequent activation of CFTR Cl^−^ channels [5, 21, 41]. Since adenosine A_2B_ receptors were found to colocalize with Ezrin, an A-kinase anchoring protein, in the luminal membranes of duct cells (Figs. 7 and 8), Ezrin may scaffold type II PKA and components of cAMP signaling pathways, including the adenosine A_2B_ receptor, adenylyl cyclase, and CFTR Cl^−^ channels [8, 12, 20, 27]. Previous studies reported that Ezrin physically interacted with type II PKA and adenosine A_2B_ receptors in intestinal epithelial cells [37]. Ezrin was also shown to associate with CFTR Cl^−^ channels by NHERF1 (also called EBP50) or NHERF2 (E3KARP) in airway epithelial cells [36, 43]. CFTR Cl^−^ channels and NHERF1/EBP50 were found to colocalize in the luminal regions of mouse pancreatic duct cells [2]. Moreover, the adenosine A_2B_ receptor physically interacted with NHERF1 in a mammalian expression system or with NHERF2 in intestinal epithelial cells [30, 37]. Furthermore, adenosine A_2B_ receptors interacted with CFTR Cl^−^ channels, which influenced the number of adenosine A_2B_ receptors in the plasma membrane [48]. A recent study reported that pancreatic ducts expressed multiple adenylyl cyclase (AC) isoforms: AC3, AC4, AC6, AC7, and AC9 [35]. Further studies are required to clarify whether Ezrin associates with adenylyl cyclase isoforms and accomplishes the compartmentalization of cAMP signaling in the luminal regions of pancreatic duct cells.

In accordance with the present results, previous studies demonstrated that adenosine A_2B_ receptors regulated Cl^−^ channels in various secretory epithelia, including airway epithelia [20], the colon [3, 42], duodenum [17], renal inner medullary collecting duct [34], middle ear epithelia [13], and CFTR-transfected CFPAC-1 cell line [33]. In addition to epithelial transport, the adenosine A_2B_ receptor is known to be involved in inflammation and immunity in the vascular system [9]. We found that adenosine A_2A_ and A_2B_ receptors were also expressed in the endothelial cells of blood vessels in the pancreas (Fig. 8d, h), which implied that these receptors may regulate blood pressure and the vascular flow rate in the pancreas [14, 51]. Furthermore, the activation of adenosine A_2B_ receptors was shown to promote the growth and metastasis of cancer [28, 40, 49]. Therefore, adenosine A_2B_ receptors may be a potential target for pancreatic cancer therapy as well as dysfunctions in epithelial transport.

Extracellular adenosine concentrations are generally considered to be less than 1 μM in unstressed tissues, whereas they may markedly increase during ischemia or inflammation [1]. Our results showed that adenosine activated anion secretion and Cl^−^ channels with *K*_d_ values of approximately 10 μM in Capan-1 cells (Figs. 1 and 6b) as well as Cl^−^ channels with a *K*_d_ value of 20 μM in guinea pig duct cells (Fig. 9d), corresponding approximately to the *K*_d_ value of 15 μM on the adenosine A_2B_ receptor [10]. In the lumen of pancreatic ducts, adenosine is produced by the hydrolysis of ATP, which acini release at 10–20 μM [38, 39, 52]. Capan-1 monolayers have also been shown to release ATP, which stimulates purinergic receptors on the luminal membrane [24]. In addition, the extracellular concentration of adenosine in supernatant collected from Capan-1 cells was 2.5 μM at basal levels [25]. Therefore, adenosine may reach high concentrations in the ductal lumen and affect adenosine A_2B_ receptors on the luminal membrane. However, we cannot rule out the contribution of adenosine A_2A_ receptors to transepithelial anion secretion and the activation of Cl^−^ channels in Capan-1 cells. Importantly, adenosine A_2A_ receptor had higher mRNA level than A_2B_ receptor did in rat pancreas. In addition, the strongest immunofluorescence ascribed to the adenosine A_2A_ receptor was detected on the luminal membrane of rat ducts [32]. A previous study proposed that adenosine A_2A_ receptors formed a functional hetero-oligomer complex with adenosine A_2B_ receptors and were involved in their surface expression [29]. Future studies are needed in order to establish the presence of the hetero-oligomer in the luminal membranes of pancreatic duct cells and the functional relevance it may have.

Electrophysiological studies on native pancreatic ductal epithelial cells have shown that 10 mM ethanol increased basal but blocked forskolin-stimulated CFTR currents [23]. We predicted that ethanol would affect the adenosine signaling and activity of Cl^−^ channels. However, ethanol (1 and 10 mM) had no effect on adenosine-stimulated conductance with gramicidin-perforated patch-clamp in guinea pig duct cells. A recent study has shown that application of ethanol had negligible effects on ATP release from Capan-1 cells [25].

In conclusion, we showed that adenosine regulated anion secretion by activating CFTR Cl^−^ channels via adenosine A_2B_ receptors on the luminal membranes of Capan-1 cells. Luminal adenosine may be another important coordinator for acini-to-duct signaling, and by virtue of supporting ductal secretion, it may help to flush out digestive enzymes delivered from acini.
